# Network Pharmacology Combined with Experimental Validation Reveals the Anti-tumor Effect of *Duchesnea indica* against Hepatocellular Carcinoma

**DOI:** 10.7150/jca.76591

**Published:** 2023-02-13

**Authors:** Xing Liu, Kesheng Wang, Luling Wang, Xingliang Fan

**Affiliations:** 1 Department of Central Laboratory Medicine, Shanghai Municipal Hospital of Traditional Chinese Medicine, Shanghai University of Traditional Chinese Medicine, Shanghai 200071, China; 2 Shanghai Tenth People's Hospital, Tongji University, School of Medicine, Shanghai, 200072, China; 3 Department of Hospital-Acquired Infection Control, Shanghai municipal Hospital of Traditional Chinese Medicine, Shanghai University of Traditional Chinese Medicine, Shanghai 200071, China

**Keywords:** *Duchesnea indica*, hepatocellular carcinoma, metabolism, tumorigenesis, angiogenesis

## Abstract

**Context:** *Duchesnea indica* is effective against hepatocellular carcinoma (HCC); however, its underlying mechanism of action remains unclear.

**Objective:** The present study aimed to investigate the potential mechanism of action and effects of *D. indica* components against HCC.

**Materials and methods:** First, the effects of *D. indica* against HCC were investigated *in vitro* and *in vivo*. For *in vitro* experiments, HCC cell lines were treated with* D. indica* solutions at different concentrations (0, 1, 2 mg/mL) and then assessed for cell apoptosis, proliferation, migration, invasion, and angiogenic ability. For *in vivo* experiments, 24 mice were randomly divided into the following four groups: model group and *D. indica* low-, medium-, and high-dose groups. Tumor growth and CD34 and Ki67 expression levels were assessed to determine the effects of *D. indica* on cell proliferation and angiogenic ability. Furthermore, transcriptome sequencing and differential expression analyses were used to identify *D. indica*-induced differentially expressed genes (DEGs) in HCC cells. Additionally, mass spectrometry was conducted to identify the chemical components of *D. indica*. Four databases were used to predict the target proteins of these chemical components in HCC. HCC-associated genes were identified from two databases. By intersecting the identified DEGs; target proteins; and HCC-associated genes, key *D. indica*-regulated HCC-related genes were identified. Subsequently, protein-protein interaction network, network pharmacology, and molecular docking were used to identify the active compounds in *D. indica* and their likely gene targets.

**Results:** *In vitro* experiments demonstrated that *D. indica* induced tumor cell apoptosis and inhibited cell proliferation, migration, invasion, and angiogenic potential. *In vivo* experiments demonstrated that *D. indica* inhibited tumor growth in a dose-dependent manner. Bioinformatic analyses identified 49 key *D. indica-*regulated HCC-related genes, of which *FOS*, *SERPINE1*, *AKR1C3*, and *FGF2* were the most significant. Mass spectrometry identified the following five molecules in *D. indica* with potential anti-HCC activity: 4′, 5, 7-trihydroxyflavone; ethyl protocatechuate; 3, 5-dihydroxy-benzoic acid; curculigosaponin A; and curculigine G. Molecular docking validated the interaction between *D. indica* active compounds and their target proteins in HCC.

**Conclusions:** The present study confirmed the therapeutic effects of *D. indica* against HCC and identified the key genes and active components that may contribute to its mechanism of action, thereby providing a basis for further research on targeted therapeutics for HCC.

## Introduction

Hepatocellular carcinoma (HCC) is a digestive tract cancer reported to cause over 2.8 million deaths in China 1. HCC is predominantly diagnosed at an advanced stage, and the survival rate is relatively poor 2. HCC is mainly treated by surgical resection and chemotherapy, which have substantially improved the recovery rate; however, the five-year survival rate after resection does not exceed 60% 3.

Since centuries, traditional Chinese medicine (TCM) has been widely applied to prevent and treat various malignant tumors in China 4, 5. TCM has gained extensive popularity owing to its excellent efficacy and low toxicity. *Duchesnea indica* (*D. indica*) is a crucial TCM recorded in various classic traditional Chinese medicine texts, such as the Compendium of Materia Medica. Over 70 compounds have been isolated from *D. indica*, including phenolic acids, phenolic esters, flavonoids, sterols, and triterpenoids 6. Further research on the active components and mechanisms of action of *D. indica* will allow a wider and more targeted application of its medicinal value. Modern pharmacological studies have shown that *D. indica* has strong anti-tumor, anti-inflammatory, anti-bacterial, and anti-hypertensive effects, as well as the ability to inhibit the central nervous system 7-10. An *in vitro* study revealed that *D. indica* extracts inhibit the migration of lung adenocarcinoma cells by suppressing epithelial-mesenchymal transition. Furthermore, the authors demonstrated that oral ingestion of *D. indica* extract leads to inhibition of tumor growth in a BALB/c nude mouse xenograft model 7. Nevertheless, the underlying molecular mechanisms of the anti-tumor effect of *D. indica* remain unknown.

Various factors contribute to the pathology of HCC, including chronic liver inflammation, chronic HBV and HCV infections, and glycometabolism 11. Owing to the complex composition of *D. indica*, the mechanism of its action against HCC remains unknown. Network pharmacology is a promising integrative approach that combines pharmacology, molecular biology, and bioinformatic tools to identify network relationships among TCM components, relevant targets, biological functions, and diseases 12. In this study, we demonstrated the anti-tumor effects of *D. indica* against HCC both *in vitro* and *in vivo*. Furthermore, we investigated the specific role of active molecules in *D. indica* through network pharmacology to determine the mechanism by which *D. indica* suppresses HCC.

## Materials and Methods

### Materials

The *D. indica* granules used in this study were obtained from Jiangyin Tianjiang Pharmaceutical Co. Ltd. (Lot number 18050481). The stock solution was prepared by dissolving *D. indica* granules at a concentration of 200 mg/mL in Dulbecco's modified Eagle medium (DMEM). The stock solution was centrifuged at 5000 rpm for 5 min at 25°C, and the supernatant was collected and frozen at -20°C.

### Cell culture

Hep3B and SK-Hep1 cell lines were used to evaluate the effects of *D. indica* on HCC tumor cells. HCT116 and L02 cell lines were used to evaluate the effects of *D. indica* on colon cancer and normal hepatocyte cells, respectively. Hep3B was purchased from the National Collection of Authenticated Cell Cultures of Chinese Academy of Sciences (Shanghai, China). HCT116 cells were purchased from Shanghai Qui Cell Biotechnology Co., Ltd. (Shanghai, China). SK-Hep1 and L02 cells were obtained from our laboratory. Hep3B cells were cultured in minimum essential medium (MEM) with 10% fetal bovine serum (FBS), 1% Glutamax (Invitrogen, 35050061), 1% non-essential amino acids (Invitrogen, 11140050), and 1% Sodium Pyruvate 100 mM solution (Invitrogen, 11360070). SK-Hep1, L02, and HCT116 cells were cultured in DMEM with 10% FBS at 37°C with 5% CO_2_.

### Detection of cell apoptosis

Hep3B and SK-Hep1 cell lines were seeded into six-well plates at a density of 2.5 × 10^5^ cells/well. The next day, *D. indica* solution was added at concentrations of 0, 1, and 2 mg/mL. After 24 h of treatment, the different cell groups were treated with annexin V-FITC/propidium iodide (KGA108, KeyGen Biotech Co. Ltd, CHN) according to the manufacturer's instructions and analyzed by flow cytometry to detect apoptosis.

### Detection of cell proliferation and colony formation

Hep3B, SK-Hep1, HCT116, and L02 cells were seeded into 96-well plates at a density of 1 × 10^4^ cells/well. The next day, *D. indica* solution was added at concentrations of 0, 1, and 2 mg/mL. The CCK-8 kit (Dojindo Laboratories, JP) was used to evaluate the effects of *D. indica* on cells after 24 h.

For the colony formation assay, Hep3B and SK-Hep1 cells were seeded into six-well plates at a density of 1 × 10^3^ cells/well, treated with 0, 1, or 2 mg/ mL of *D. indica* solution, and allowed to adhere for 14 days. The cells were stained with 0.1% crystal violet, photographed, and counted.

### Detection of cell migration and invasion

A wound-healing assay was performed to evaluate cell migration ability. Hep3B and SK-Hep1 cells were seeded into six-well plates at a density of 3 × 10^5^ cells/well. The next day, after agitating with 200 µL pipette tips, *D. indica* solution was added at concentrations of 0, 1, and 2 mg/mL. Images were obtained every 12 h for the following 24 h.

For cell invasion assays, iced Matrigel (356234, Corning Inc., USA) was mixed with DMEM at a ratio of 1:4, and a 50 µL mixture per well was added to the upper layer of the chamber (356234, Corning Inc., USA) and solidified at 37°C. Serum-free medium containing *D. indica* solution at concentrations of 0, 1, and 2 mg/mL was added to the upper chamber with 1 × 10^5^ cells, and 500 µL DMEM was added to each well of the lower chamber. The chamber was then placed in a cell culture box for 72 h. After wiping the Matrigel, the cells were stained with 0.1% crystal violet, photographed, and counted.

### Detection of tube formation

First, 50 µl iced Matrigel (356234, Corning Inc., USA) was added to the bottom of a 96-well plate. After solidification at 37°C, 100 µl of medium containing 2 × 10^5^ human umbilical vein endothelial cells was placed into each well, and *D. indica* solution was added at concentrations of 0, 1, and 2 mg/ mL. After culturing for 5 h and 20 h, the cells were observed and photographed.

### Detection of tumorigenesis and angiogenesis in nude mice *in vivo*

Male BALB/c nude mice aged 4-6 weeks were purchased from Shanghai SLAC Laboratory Animal Co., Ltd. (Shanghai, China) and housed in appropriate animal care facilities for the duration of the experimental period. Experiments were performed under a project license (SHDSYY-2018-1984) granted by the institutional ethics board of the Shanghai Tenth People's Hospital, in compliance with the Shanghai Tenth People's Hospital Institutional Guidelines for the Care and Use of Animals.

To establish a subcutaneous tumor xenograft model, 5 × 10^6^ Hep3B cells in 100 μL phosphate-buffered saline (PBS) were inoculated into the limbs of male mice weighing 15‒20 g. A total of 24 nude mice were randomly divided into four groups (model, low-, medium-, and high-dose groups, n = 6 per group). During inoculation, the nude mice were disinfected with alcohol cotton balls and inoculated subcutaneously under the armpit. After inoculation, nude mice were caged separately according to their treatment group and regularly fed with water and food. Tumor size was measured every seven days after tumor formation. Subsequently, the low-, medium-, and high-dose groups were treated with 0.195 g/kg, 0.39 g/kg, and 0.78 g/kg of *D. indica,* respectively, for three weeks. These dosages were calculated according to the clinical routine dosage of *D. indica* granules (3 g/70 kg for adults), with reference to the Methodology of Pharmacological Experiment (fourth Ed) 13.

After treatment, mice were sacrificed and photographed. The tumors were then removed and weighed. HE staining was performed to detect the internal morphology of the tumors, and Ki67 and CD34 expression levels were detected by immunohistochemistry. To investigate whether *D. indica* caused side effects on the animals, their livers and kidneys were obtained and weighed. HE staining was also conducted to detect the cell morphology of the liver and kidney tissues.

### Transcriptome sequencing

Hep3B cells treated with 1 mg/mL *D. indica* and untreated Hep3B cells were used as the treatment (M1) and control groups (M0), respectively. RNA extraction was performed using the FastPure Cell/Tissue Total RNA Isolation Kit (Vazyme, RC101). Furthermore, a cDNA library was constructed using rRNA removal and a chain-specific library scheme. The Ribo-off rRNA Depletion Kit (Human/Mouse/Rat, N406) (Vazyme) was used for rRNA removal. cDNA transcription and chain-specific library construction were carried out using the VAHTS Universal V6 RNA-seq Library Prep Kit for Illumina (NR604, Vazyme). The Illumina NextSeq 500 high-throughput sequencing platform with a high-output (300 cycles) sequencing reagent was used for sequencing.

### Identification of differentially expressed genes (DEGs)

Differential expression analysis was conducted between M0 and M1 groups. Hisat 14 was used for alignment of RNA clean data to obtain read genomic information, and HTSeq-count python script 15 was used for read counting to obtain the expression abundance of each gene. The following filtering was performed: single genes with more than 10 reads were retained; three of the six samples exceeding 10 reads were considered to be detected. The subsequent differential expression analyses were conducted with a threshold of false discovery rate (FDR) < 0.05 and |log fold change (FC)| > 1. Gene Set Enrichment Analysis (GSEA) was performed to identify significantly enriched pathways in the control and treatment groups. Parameters were set as number of permutations: 1000; permutation type: gene set.

### Mass spectrometry (MS) for the identification of chemical compounds

*D. indica* granules were dissolved in PBS solution, centrifuged at 12000 rpm for 10 min, and the supernatant was taken for MS analysis. Ultra-high performance liquid chromatography-quadrupole time-of-flight MS was used to collect data. The reverse-phase column used was a Kinetex XB-C18 column (100 mm × 2.1 mm, 2.6 μm, Phenomenex). The flow rate was set at 0.35 mL/min. The mobile phase consisted of solvent A (0.1% formic acid in aqueous solution, v/v) and solvent B (0.1% formic acid in acetonitrile solution, v/v). The elution gradients were 0.0-1.0 min, 1% B; 1.0-8.0 min, 5%-85% B; 8.0-12.0 min, 85% B; 12.0-12.1 min, 85-5% B; and 12.1-15, 5% B.

An AB-SCIEX Triple TOF 4600 high-resolution MS system was used to collect data in the electrospray ionization (ESI)-positive and ESI-negative modes. The MS conditions were as follows: collision energy, 40±15(+ESI), -40±15(-ESI); ion spray voltage: cationic mode +5500 V, anionic mode -4500 V; atomizer temperature: 600°C; atomizing gas pressure: 55 psi; dry gas pressure: 55 psi; air curtain air pressure: 25 psi; full scan analysis; quality scan range: 50-1200 Da. The frequency of primary MS was 0.25 s and that of secondary MS was 0.1 s. An IDA acquisition mode was used to collect data, with eight data each time.

Analyst TF 1.6.2 software was used to collect data. PeakView 1.5.1 was used for data extraction to obtain a 3D matrix of raw data, including the compound charge retention time and ion abundance. OSI-SMMS software (Dalian Dashuo Bioinformation Technology Co. Ltd.) was used to preprocess the raw data. Compounds were screened based on the Human Metabolome Database (HMDB) and MassBank databases and finally confirmed by comparing the secondary MS with the standard spectrum in the database.

### Identification of compound-target proteins through network analysis

Based on the 22 chemical compounds identified in the MS analysis, we predicted the target proteins using the Traditional Chinese Medicine Systems Pharmacology Database and Analysis Platform (TCMSP) 16, Batman database 17, SwissTargetPrediction online prediction software 18, and PharmMapper Server online prediction software 19. The threshold in the Batman database was designed with a cutoff score of 20 and an adjusted p-value of 0.05. The threshold in SwissTargetPrediction was designed with a probability > 0. The thresholds for the rest of the database/prediction software programs were set to default.

The overlapping proteins obtained by intersecting the target proteins from the four databases were defined as the targets of *D. indica* components. The network of component-target proteins was constructed using Cytoscape 20 (Version 3.6.1). The CytoNCA 21 plug-in was used to perform Degree Centrality (DC) analysis on nodes in the network, and the parameter was set to the width weight. The key components were obtained by ranking the connectivity of each node.

### Identification of target genes associated with HCC

Genes associated with HCC were searched in the GeneCard 22 databases using “liver cancer,” “hepatocellular carcinoma,” and “hepatoma” as keywords. Simultaneously, genes associated with HCC were also searched in the Comparative Toxicogenomics Database (CTD) 23 with “carcinoma” and “hepatocellular” as the keywords. Subsequently, disease-related genes were intersected with the DEGs and small molecular target proteins to obtain key genes associated with HCC and targeted by small molecules.

Subsequently, the clusterProfiler 24 package was used to explore the biological process (BP) and KEGG pathway in which disease-associated target genes were enriched. SimplifyEnrichment 25 was used to generate the BP results owing to its massive terms in BP.

### PPI network construction

A protein-protein interaction (PPI) network was established using the STRING (version 11.0) database to further investigate the interaction relationships among the key intersecting genes 26. The species was selected as “homo sapiens.” Cytoscape was used to construct the network diagram. The CytoNCA plug-in was used to analyze the connection degree of the nodes in the PPI network. Important nodes involved in the PPI network were obtained using the connection degree ranking of each node.

Subsequently, cluster analysis was performed to identify the functional modules in the PPI network. The plug-in MCODE 27 (version 1.5.1) in Cytoscape was used to identify the significant modules or protein complexes with biological significance and screen the most crucial genes in the PPI network. The parameters were set to default (Degree Cutoff = 2, Node Score Cutoff = 0.2, K-core = 2, Max. Depth = 100).

### Construction of network pharmacology

From the aforementioned analysis, the relationship between the component-target protein and the target protein pathway was constructed. By integrating these relationships, Cytoscape was used to construct network pharmacology, exhibiting the pathway regulation mechanism of relevant target proteins targeted by key *D. indica* components.

### Molecular docking for the validation of compound‑target interactions

Molecular docking was conducted to explore the interactions between the crucial compounds and their targets. The structures of the active compounds and their targets were obtained from the Protein Data Bank 28 (PDB) and PubChem 29, respectively. The downloaded structures were converted into PDB format for molecular docking. AutoDock 30 (Version 4.2.6) was used to investigate putative docking between the molecules and targets. The Lamarckian GA algorithm was used to conduct molecular docking.

### Statistical analysis

All analyses were performed using R software (version 3.6.2), GraphPad Prism 7.0, and SPSS 22.0. Cell invasion was measured using the Image-Pro Plus 6.0 software. The parameters of tube formation and tumor staining data were measured using ImageJ software. The statistical significance of the differences among tumor sizes was calculated using a two-way analysis of variance. The significance of other differences was measured using a two-tailed student's t-test. p < 0.05 was considered significant.

**Figure 1:**
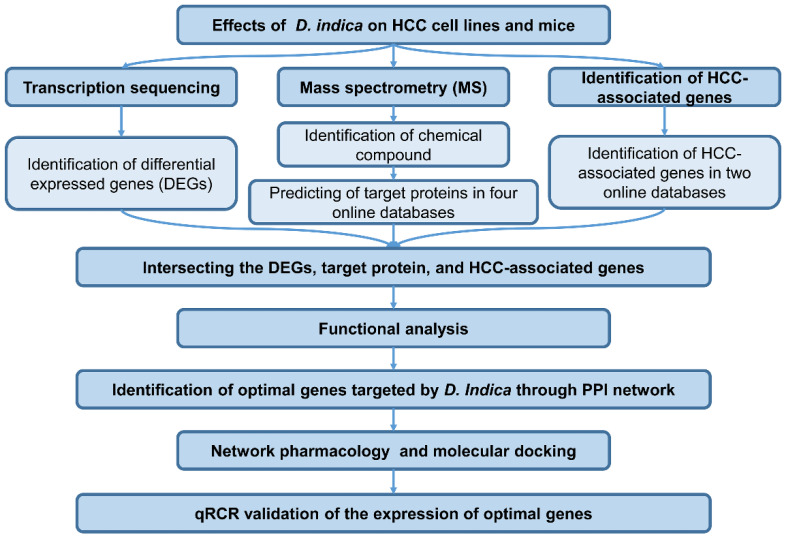
Workflow of this study. HCC, hepatocellular carcinoma.

The workflow of this study is shown in Figure 1.

## Results

### D. indica induces cell apoptosis and inhibits cell proliferation in HCC cells

**Figure 2:**
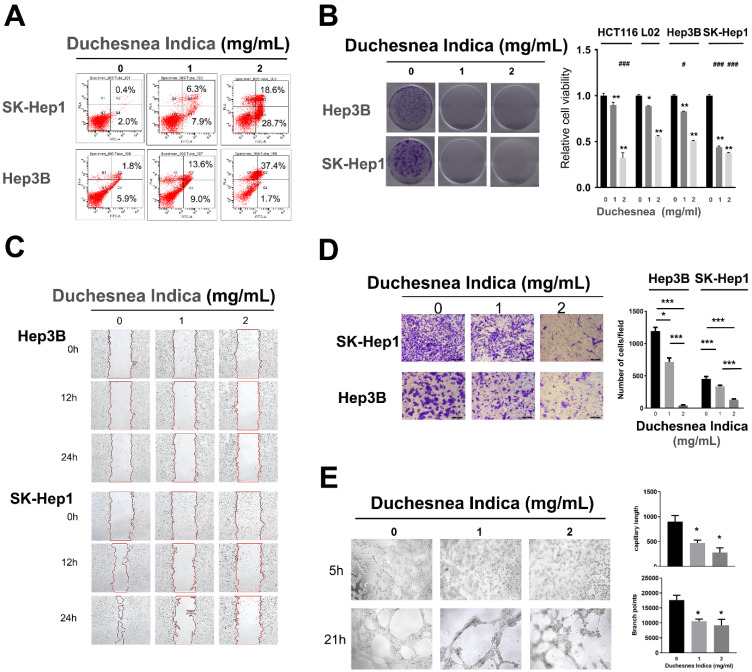
The effects of *D. indica* on HCC cancer cells. (A) *D. indica* induces apoptosis. (B) *D. indica* inhibits cell proliferation. Left, results of colony formation assay; right, results of CCK-8 assay. (C) *D. indica* inhibits cell migration. (D) *D. indica* suppresses tumor cell invasion. (E) *D. indica* decreases angiogenic ability. *, significant differences between cells with and without *D. indica*; *, p < 0.05; **, p < 0.01; ***, p < 0.001. #, significant differences between L02 normal cells and other cancer cells; #, p < 0.05; ##, p < 0.01; ###, p < 0.001.

To assess the effect of *D. indica* on HCC cells *in vitro*, we treated Hep3B and SK-Hep1 cell lines with *D. indica* solution. We observed an evident increase in the apoptosis rate following treatment in both cell lines, indicating that *D. indica* might induce apoptosis in HCC cells (Figure 2A).

Treatment with *D. indica* solution also significantly reduced cell viability in a dose-dependent manner in both Hep3B and SK-Hep1 cells (Figure 2B, p < 0.05), thereby indicating that *D. indica* can inhibit HCC cell proliferation.

### D. indica inhibits cell migration, invasion, and angiogenesis in HCC cells

In SK-Hep1 cells, the control group exhibited a high migration rate over time, whereas *D. indica* treatment inhibited this migration, indicating that *D. indica* might inhibit cell migration in HCC patients (Figure 2C).

In both Hep3B and SK-Hep1 cell lines, *D. indica* treatment significantly inhibited the invasion rate in a dose-dependent manner (p < 0.05 compared to control group), indicating that *D. indica* might also suppress tumor cell invasion (Figure 2D).

We also assessed the effect of* D. indica* on angiogenesis using a tube formation assay. This revealed that *D. indica* treatment decreased both the capillary length and branch points, suggesting that *D. indica* may inhibit the angiogenic ability of HCC cells (Figure 2E, p < 0.05).

### D. indica inhibits cell proliferation in a colon cancer cell line

All the above evidence confirmed the inhibitory effects of *D. indica* on HCC cells. To further investigate the effects of *D. indica* in other cancer cells, as well as non-transformed cells, we investigated the effects of *D. indica* on cell proliferation in the colon cancer cell line HCT116 and the normal hepatocyte cell line L02. The results showed that *D. indica* exerted significant inhibitory effects in the colon cancer cell line (Figure 2B). Although *D. indica* also significantly inhibited proliferation in normal hepatocyte cells, the effect was less pronounced than that seen in HCC cells, particularly at higher doses of *D. indica*.

### D. indica inhibits tumor growth and angiogenesis in HCC mice

**Figure 3:**
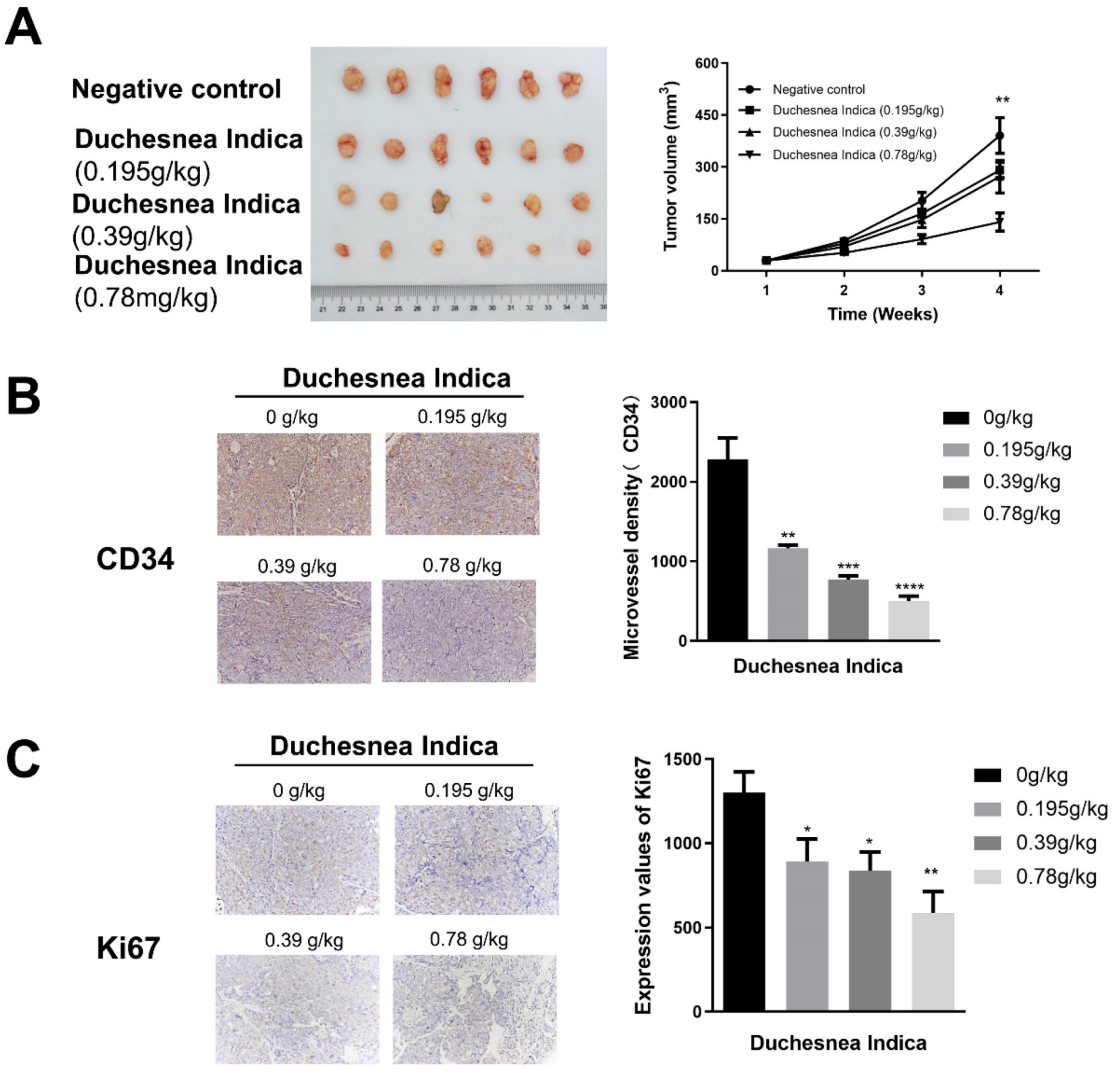
The effects of *D. indica* on tumorigenesis in HCC xenograft mice. (A) The high-dose treatment group exhibited lower tumor size. (B) *D. indica* solution decreased angiogenic potential. (C) *D. indica* solution decreased tumor proliferation ability. *, p < 0.05; **, p < 0.01; ***, p < 0.001; ****, p < 0.0001.

We next investigated the effect of *D. indica* on tumorigenesis *in vivo* by treating HCC xenograft model mice with a low, medium or high dose of *D. indica* solution. Following tumor growth, we assessed tumor volume as well as CD34 and Ki67 expression levels to indicate the angiogenic and tumor proliferation abilities, respectively. Treatment with *D. indica* led to a decrease in tumor size, which was most significant in the high-dose treatment group (Figure 3A, p < 0.01). Treatment with *D. indica* also significantly decreased the expression levels of CD34 and Ki67 (Figure 3B and 3C, p < 0.05), further demonstrating its anti-angiogenic and anti-tumor potential in HCC. Furthermore, all of these effects were dose-dependent, with higher concentrations of *D. indica* solution resulting in smaller tumor size and lower CD34 and Ki67 expression levels.

### D. indica exhibits no side effects in mice

To exclude the possibility that *D. indica* causes unwanted side effects in mice, we measured the weight of their liver and kidneys; however, no significant change was observed following *D. indica* intervention compared with controls (Figure S1A). Furthermore, HE staining in the liver and kidney tissue showed normal cell morphology with no necrosis or inflammatory cell infiltration (Figure S1B), thereby indicating that *D. indica* was well-tolerated in mice at the doses used in this study.

### Identification of DEGs

**Figure 4:**
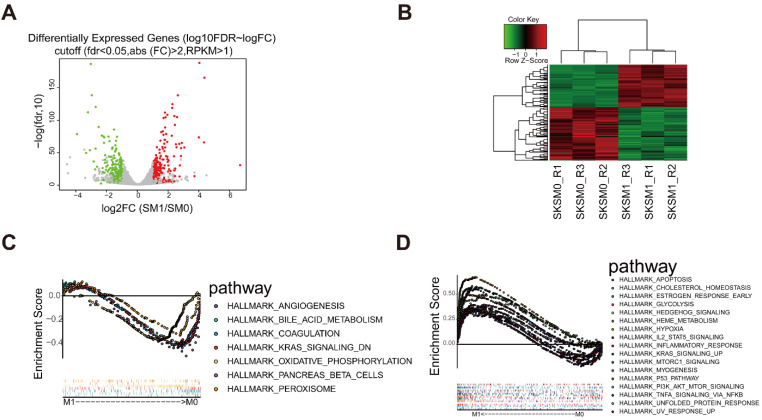
Identification of differential expressed genes (DEGs) following *D. indica* treatment of HCC cells. (A) Volcano plot of the identified DEGs. The red, green, and grey dots represent genes which were upregulated, downregulated, and not significantly changed, respectively. (B) Heat plot depicting the expression levels of the DEGs. (C-D) The pathways significantly enriched in the (C) control (without *D. indica*) and (D) treatment groups (with *D. indica*).

To further investigate the mechanism of action of *D. indica,* we analyzed the related changes in gene expression. We used Hep3B cells treated with 1 mg/mL *D. indica* and untreated Hep3B cells as the treatment (M1) and control groups (M0), respectively. According to the transcriptome sequencing results, we obtained 1017 DEGs with a threshold of FDR < 0.05 and |logFC| > 1, of which 417 and 600 genes were upregulated and downregulated, respectively (Figure 4A and B). The expression of these DEGs were significantly different between the M0 and M1 groups, thereby indicating that these genes were affected by *D. indica* intervention and might be potential target genes. GSEA analysis suggested that the DEGs were enriched in seven and 17 pathways in the M0 and M1 groups, respectively (Figure 4C and D). These included pathways such as angiogenesis, KARS signaling, apoptosis, the inflammatory response and P53 pathways, all of which are important in HCC progression.

### Identification of compound-target proteins through network analysis

**Figure 5:**
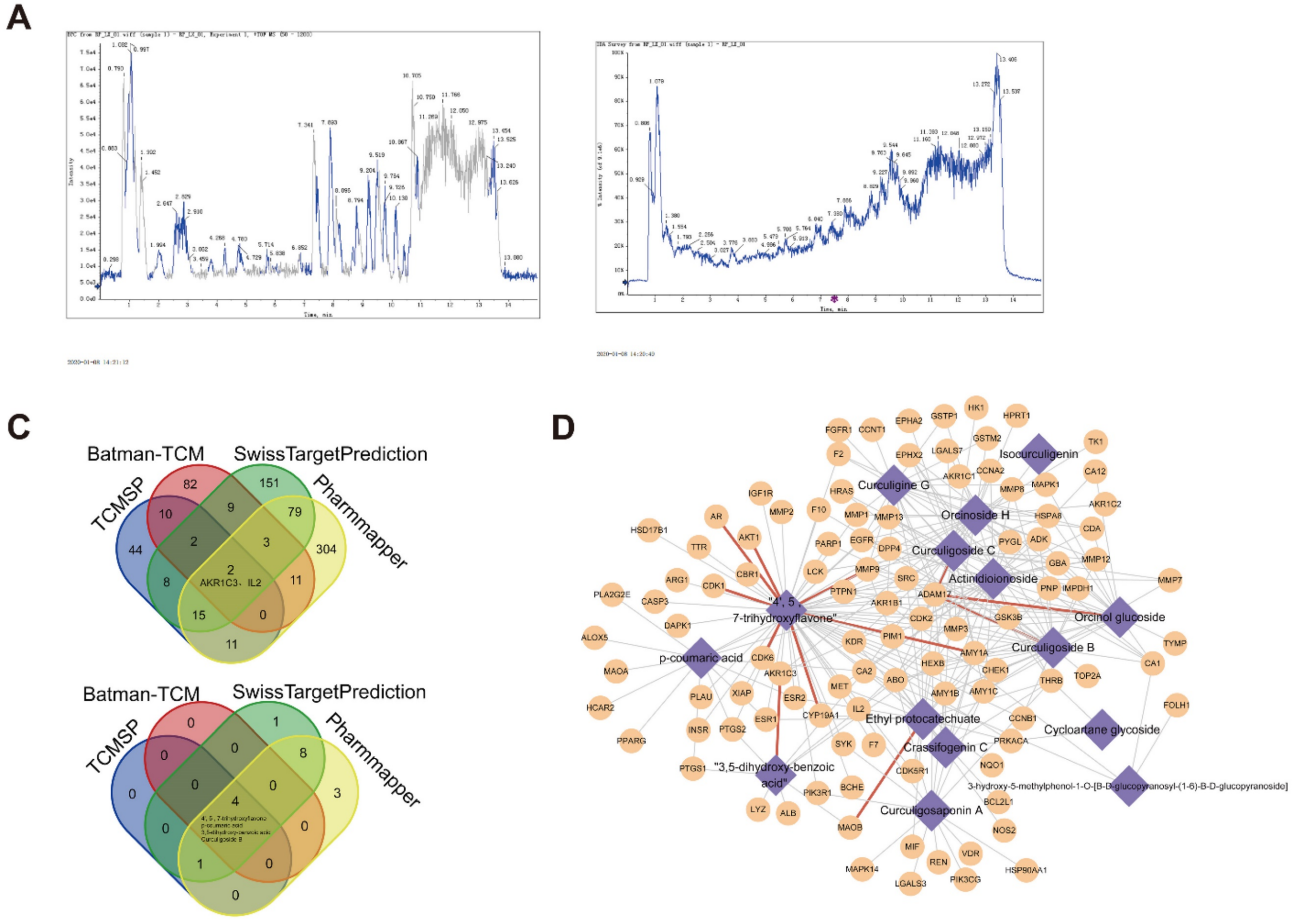
Identification of active component molecules of *D. indica* and their target proteins. (A) The results of mass spectrometry. (B) Intersection analysis of target proteins and their active components in *D. indica* based on four databases. (C) Network of the target proteins and active components.

We next performed MS analysis on *D. indica* extract to elucidate its molecular composition and identify potential target proteins. The MS analysis revealed 22 chemical molecules (Figure 5A). Database searching for potential targets revealed a total of 92, 119, 269, and 425 target proteins from TCMSP, Batman-TCM, SwissTargetPrediction, and Pharmmapper, which targeted 5, 4, 14, and 16 chemical molecules, respectively. Intersection analysis identified two target proteins (AKR1C3 and IL2) and four chemical molecules (4′, 5, 7-trihydroxyflavone, p-coumaric acid, 3, 5-dihydroxy-benzoic acid, and curculigoside B) in the four databases (Figure 5B).

Integration of the previous analyses led to the identification of 4618 molecule-target protein relationship pairs, including 17 molecules and 731 target proteins. The number of targets corresponding to each small molecule is presented in Table 1. Owing to the large number of relationship pairs, we further determined the occurrence frequency of these related pairs in the four databases. As shown in Figure 5C, 283 related pairs appeared in at least two databases. These results demonstrated a tight correlation between these molecules and target proteins.

### Identification of HCC-associated potential D. indica target genes

**Figure 6:**
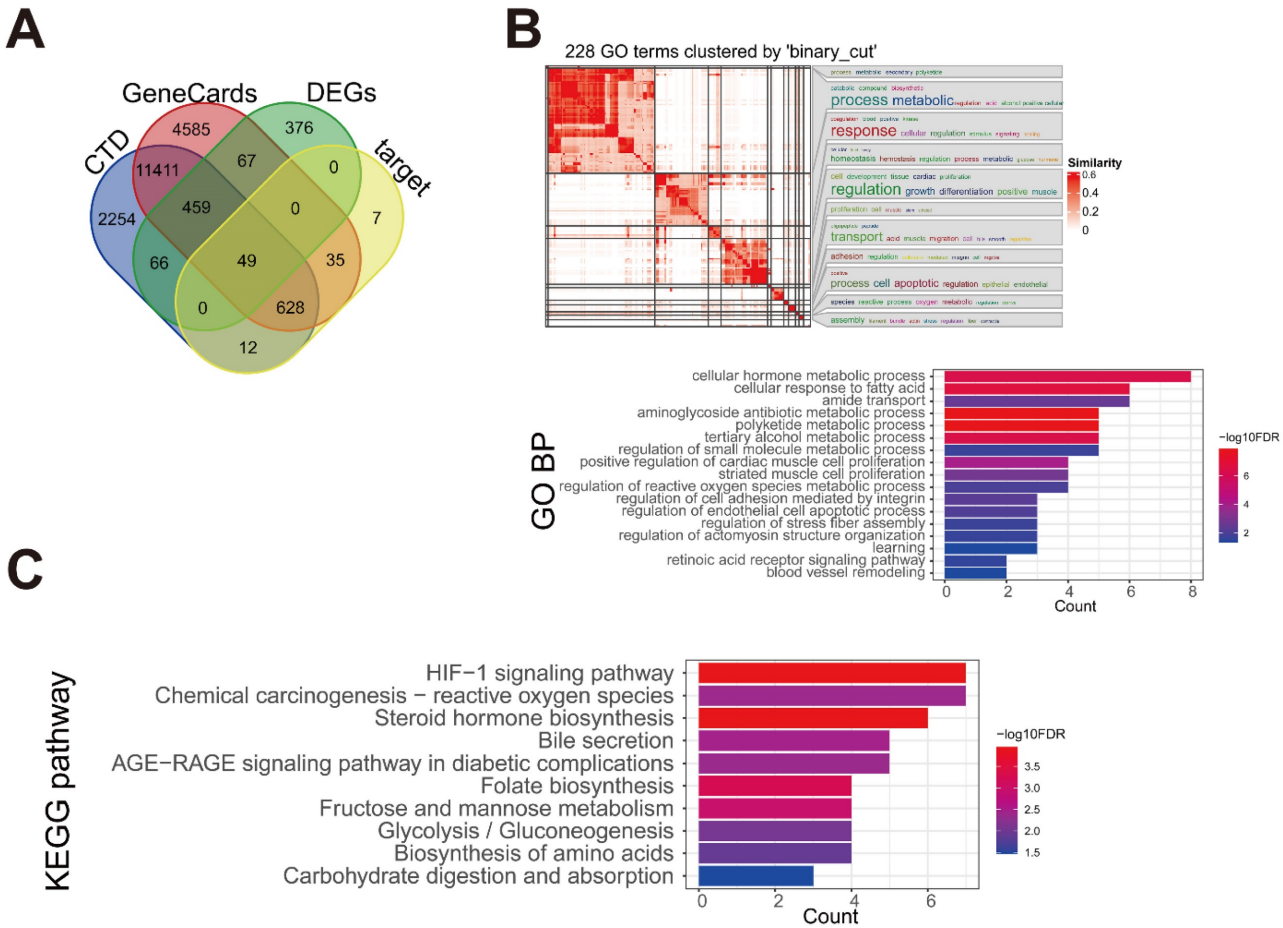
Identification of the key genes associated with HCC. (A) The intersection Venn diagram of DEGs, target proteins, and HCC-associated genes from two databases. (B) The enriched Gene Ontology-biological process (GO-BP) of the key genes. (C) The enriched Kyoto Encyclopedia of Genes and Genomes (KEGG) pathways of the key genes.

A total of 17234 and 14879 HCC-related genes were obtained from the GeneCards and CTD databases, respectively. By intersecting the HCC-related genes with the 1017 DEGs and 731 target proteins identified in our study, 49 key disease-associated genes were identified (Figure 6A). These genes were enriched in 228 BP terms and 10 KEGG pathways. Further generation analysis revealed 17 BP clusters, and the most significant terms in each cluster were visualized (Figure 6B). Disease-related genes were involved in BP terms related to cellular metabolic processes, cellular responses to fatty acids, cell proliferation, and cell apoptosis. The 10 KEGG pathways are shown in Figure 6C. The enriched pathways included the hypoxia-inducible factor 1 (HIF-1) signaling pathway, chemical carcinogenesis-reactive oxygen species, steroid hormone biosynthesis, bile secretion, and the AGE-RAGE signaling pathway, which is associated with diabetes.

### Identification of important D. indica-regulated genes in HCC through PPI network analysis

**Figure 7:**
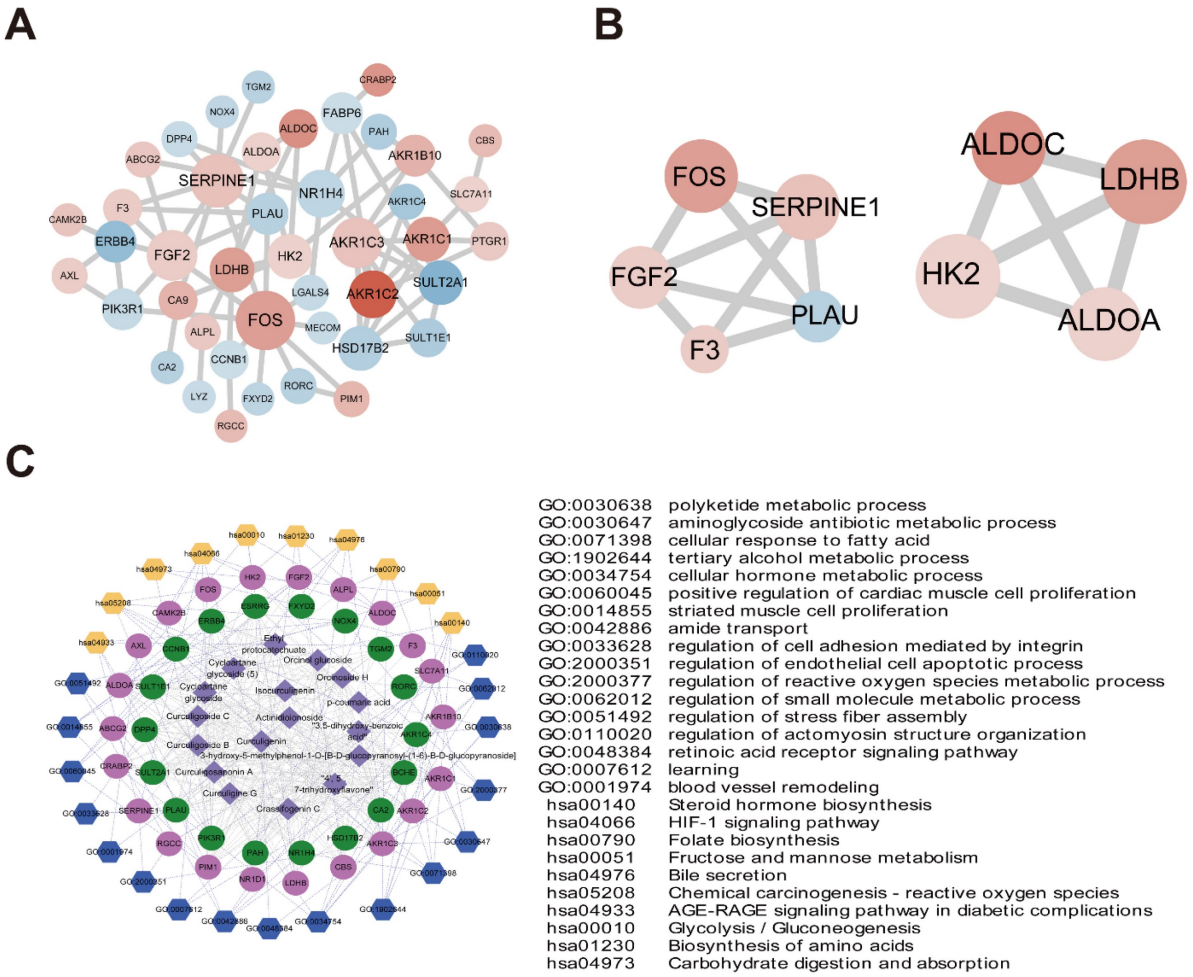
PPI networks and network pharmacology. (A) PPI network diagram of 49 key genes (red and blue indicate up- and downregulation after *D. indica* treatment, respectively, and a darker color indicates greater absolute difference multiple). (B) Two submodules mined in PPI. (C) Compound small molecules-disease key genes-functional/pathway network diagram (the purple diamond represents small molecule compounds; the red and green circles represent the up- and downregulation of key genes, respectively. The blue and yellow hexagons represent enrichment of the GO-BP and KEGG pathways, respectively. The table on the right shows specific contents of the corresponding GO-BP or KEGG pathway; the gray line represents protein relationships of target genes and small chemical molecules; the light purple line represents the pathway or function of genes).

The 49 *D. indica-*regulated, HCC-related genes identified above were input to the STRING online database, and a PPI network with 43 nodes was established after removing discrete proteins. The PPI network contained 70 protein-interaction pairs (Figure 7A). In the connection degree analysis, the top four key genes, *FOS*, *SERPINE1*, *AKR1C3*, and *FGF2* (*AKR1C3* and *FGF2* showed the same connection degree), exhibited strong connections with other genes in the PPI network, suggesting a crucial role in the activity of *D. indica* against HCC. Module analysis screened two important modules in the PPI network (Figure 7B), of which the top three genes, *FOS*, *SERPINE1*, and *FGF2*, in module one were demonstrated to be important in HCC.

### Network pharmacology analysis of the active component-key target protein-pathway network

As described previously, an active component-key target protein-pathway network with 745 relationship pairs was established. This network contained 17 compounds, 40 target proteins, 17 BP terms, and 10 KEGG pathways (Figure 7C). The main functions of active compound-targeted proteins were associated with metabolic processes, cell proliferation, adhesion, and apoptosis.

### Validation of compound‑target interactions

**Figure 8:**
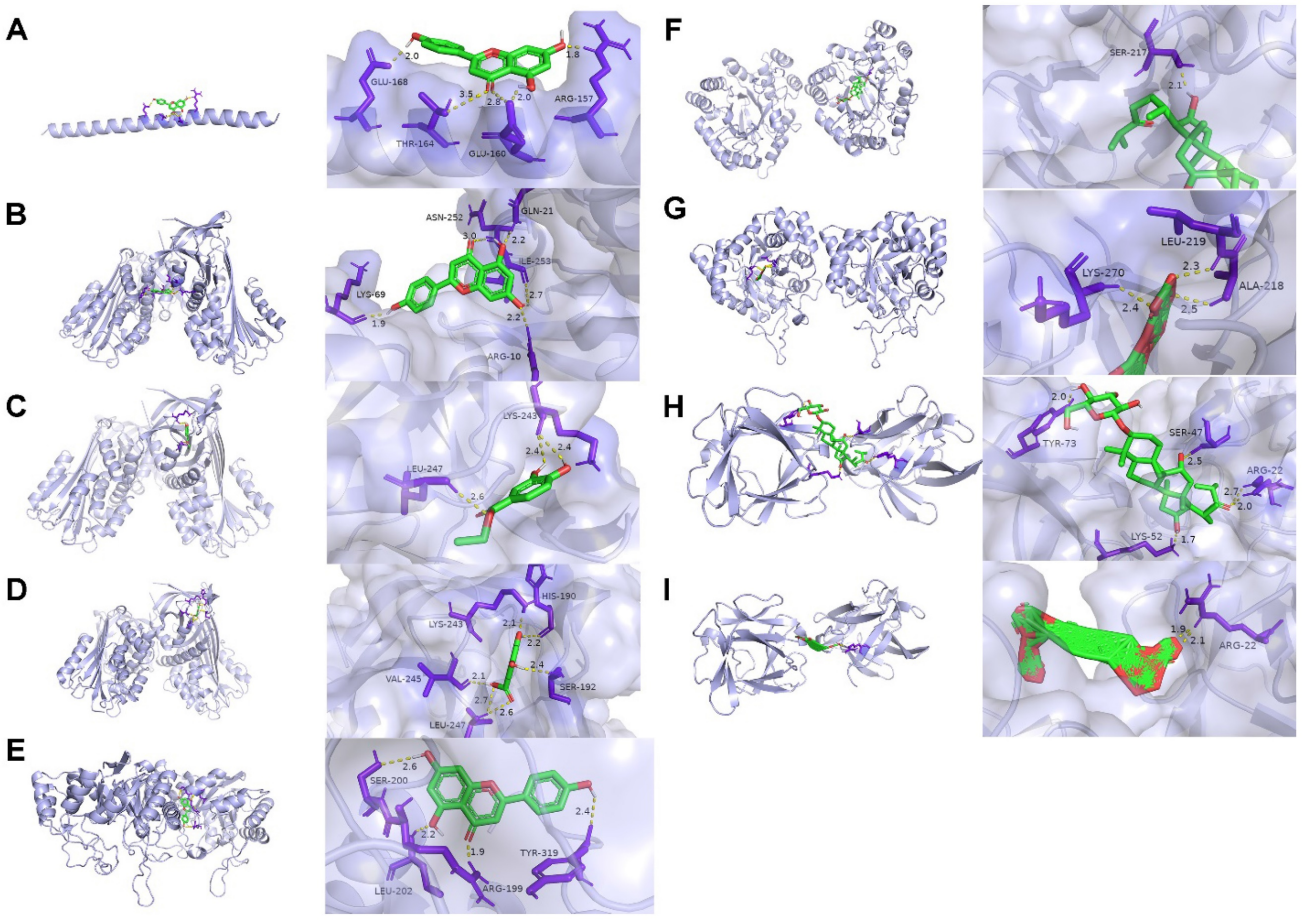
Molecular docking results. (A) 4′, 5, 7-trihydroxyflavone targeting FOS. (B) 4′, 5, 7-trihydroxyflavone targeting SERPINE1. (C) Ethyl protocatechuate targeting SERPINE1. (D) 3, 5-dihydroxy-benzoic acid targeting SERPINE1. (E) 4′, 5, 7-trihydroxyflavone targeting AKR1C3. (F) Curculigosaponin A targeting AKR1C3. (G) Curculigine G targeting AKR1C3. (H) Curculigosaponin A targeting FGF2. (I) Cycloartane glycoside (5) targeting FGF2. The light purple, green, yellow, and dark purple represent the protein receptor, compound small molecule ligand, hydrogen bond interaction between the protein receptor and compound small molecule ligand, and amino acid residue with hydrogen bond interaction with the compound small molecule, respectively.

The top three genes in the PPI network were selected as the optimal target protein genes, and their corresponding drug molecules were screened for molecular docking. Nine relationship pairs were selected for molecular docking (Table 2 and Figure 8). These results further validated the interacting roles of the compounds and their target proteins.

### Validation of the D. indica-induced expression of key target genes

To further investigate the potential expression changes of *FOS*, *SERPINE1*, *AKR1C3*, and *FGF2* after *D. indica* treatment, we conducted qPCR analysis. The results suggested that *D. indica* treatment increased the expression levels of *FOS*, *SERPINE1*, *AKR1C3*, and *FGF2* (Figure S2), which further demonstrated their important role in HCC.

## Discussion

TCM has been effective in treating various tumors in China since many centuries 4, 5. *D. indica* is one of the most important TCMs, and exhibits strong anti-tumor, anti-inflammatory, anti-bacterial, and anti-hypertensive effects, as well as the ability to inhibit the central nervous system 7-10. Nevertheless, the underlying molecular mechanism of the inhibitory role of *D. indica* remains unknown.

In this study, we conducted *in vitro* cell and *in vivo* mouse experiments to explore the role of *D. indica* in HCC. The results showed that *D. indica* can induce tumor cell apoptosis, inhibit cell proliferation, migration, and invasion, and attenuate tumor growth and angiogenic potential. Moreover, the inhibitory role of *D. indica* was also validated in colon cancer cells. The results of this study were consistent with those of previous studies 31, 32. Although *D. indica* exerts an inhibitory effect on non-transformed cells, it is less pronounced than that observed in cancer cells. Moreover, *D. indica* exhibited no significant side effects on the livers or kidneys of animals in our mouse experiments.

In the subsequent bioinformatic analyses, we aimed to explore the key genes and active components of *D. indica* that regulate its anti-tumor role. A total of 1017 DEGs were identified between treatments with and without *D. indica*, of which 417 were upregulated and 600 were downregulated. MS analysis revealed 22 chemical molecules in *D. indica*, 17 of which gave a total of 731 target proteins identified in four databases. Two target proteins (AKR1C3 and IL2) and four chemical molecules (4′, 5, 7-trihydroxyflavone; p-coumaric acid; 3, 5-dihydroxy-benzoic acid; and curculigoside B) were found in all four databases. Further analysis showed a close relationship between the *D. indica* compounds and their target proteins. Subsequently, the target proteins, DEGs, and HCC-related genes were intersected, and 49 key disease-associated genes were identified. These genes were enriched for functions related to metabolic processes, cell proliferation, and cell apoptosis. These genes were also enriched in pathways related to chemical carcinogenesis, steroid hormone biosynthesis, and bile secretion. This evidence demonstrates that these genes are closely associated with the development of malignant tumors. PPI analysis showed that *FOS*, *SERPINE1*, *AKR1C3*, and *FGF2* were the most important genes associated with *D. indica* in terms of HCC. Network pharmacology analysis showed that the main functions of active compound-targeted proteins were associated with metabolic processes, cell proliferation, cell adhesion, and cell apoptosis. Molecular docking further validated the interaction between the active compounds and targeted proteins.

Our results confirmed the inhibitory effects of *D. indica* on tumors. *FOS*, *SERPINE1*, *AKR1C3*, and *FGF2* are the genes that potentially play important roles in HCC and are associated with tumor growth. The FOS family includes c-Fos, FosB, Fra1, and Fra2 33. c-Fos was highly expressed in patients with HCC 33, 34. Bioinformatics also demonstrated the prognostic potential of FOS, with higher FOS expression levels indicating lower overall survival 35. *FOS* gene expression is related to cell proliferation, differentiation, transformation, and apoptosis 36, and liver carcinogenesis is related to *FOS*-dependent inflammation 33. In addition, FOS forms an AP-1 transcription factor complex, which dimerizes with the JUN family of proteins. SERPINE1 is upregulated in liver cancer cell lines, and its high expression accelerates cancer 37. *SERPINE1* variation also plays an important role in defining therapeutic outcomes in patients with HCC 38. *AKR1C3* catalyzes the conversion of aldehydes and ketones to alcohols and plays an important role in multiple cancers. High expression of AKR1C3 is associated with a worse prognosis in patients with HCC, and knockdown of *AKR1C3* inhibits tumor cell proliferation, decreases cell viability, and inhibits tumorigenesis 39. FGF2 is the most potent angiogenic factor. FGF2 plays an important role in tumor growth, and targeting FGF2 inhibits tumor angiogenesis 40. Repressing expression of FGF2 can inhibit cancer cell proliferation and metastasis in lung cancer 41. The above evidence suggests that *FOS*, *SERPINE1*, *AKR1C3*, and *FGF2* are important in the progression of HCC. Considering that these genes are important target genes of *D. indica*, they may also be crucial for the therapeutic effect of *D. indica* on HCC. Functional analysis in this study also showed the important role of these genes in cancer progression and biological metabolism. Finally, qPCR indicated that these genes may be differentially expressed in *D. indica*-treated cells as compared with untreated cells.

Network pharmacology analysis demonstrated that the active targeting compounds in *D. indica* might be 4′, 5, 7-trihydroxyflavone; ethyl protocatechuate; 3, 5-dihydroxy-benzoic acid; curculigosaponin A; and curculigine G. The compound 4′, 5, 7-trihydroxyflavone, also named apigenin, is a natural flavonoid with low intrinsic toxicity. Apigenin has been shown to possess potential pharmacological effects against HCC 36. The inhibitory role of apigenin in HCC cells may be mediated by the upregulation or downregulation of miRNA molecules and their related target genes, such as *FOS* 36. A study conducted in hamsters also suggested that apigenin induces disorders in cell proliferation, apoptosis, angiogenesis, and inflammatory markers 42. Ethyl protocatechuate was reported to induce cell autophagy and apoptosis through the upregulation of *BNIP3* and *NDRG1* (N-myc downstream-regulated gene-1) in esophageal squamous cell carcinoma cells 43. The roles of 3, 5-dihydroxy-benzoic acid; curculigosaponin A; and curculigine G in cancer remain unknown. These results demonstrate that the identified active components in *D. indica* might be important mediators of some of the target genes identified, although further clarification of this mechanism is undoubtedly required.

The *in vitro* expriments in the present study suggested that *D. indica* induced tumor cell apoptosis and inhibited cell proliferation, migration, and invasion. *In vivo* analysis also suggested that *D. indica* attenuated angiogenic potential and tumor growth. We also showed that the key genes and active molecules involved in the action of *D. indica* against HCC are associated with metabolic processes and cell proliferation, adhesion, and apoptosis. Nevertheless, this study has some limitations. First, the key genes identified were only validated in the cell lines; therefore, further research is needed to confirm these findings in animal models and clinical samples. Second, the specific roles of active molecules, as well as their correlation with the key genes, were not confirmed in this study. Finally, more work needs to be conducted to explore the interactions of genes, molecules, and biological processes.

## Conclusion

In this study, *in vitro* and *in vivo* experiments confirmed the important role of *D. indica* in suppressing tumor growth by inducing tumor cell apoptosis and inhibiting cell proliferation, migration, invasion, and angiogenesis. Further bioinformatics analyses identified four important genes (*FOS*, *SERPINE1*, *AKR1C3*, and *FGF2*) and several active molecules (4', 5, 7-trihydroxyflavone; ethyl protocatechuate; 3, 5-dihydroxy-benzoic acid; curculigosaponin A; and curculigine G) with potential therapeutic roles in the activity of *D. indica* against HCC. Network pharmacology and molecular docking studies have indicated that these genes and compounds are important in biological metabolism and cell growth.

## Supplementary Material

Supplementary figures and table.

## Figures and Tables

**Table 1:** The number of targets corresponding to each small molecule

Molecule	Target count
4', 5, 7-trihydroxyflavone	422
Curculigosaponin A	334
Curculigine G	333
Ethyl protocatechuate	329
Actinidioionoside	325
Orcinoside H	323
Curculigoside C	319
Curculigoside B	313
Orcinol glucoside	311
3, 5-dihydroxy-benzoic acid	290
Cycloartane glycoside	289
Crassifogenin C	288
p-coumaric acid	232
3-hydroxy-5-methylphenol-1-*O*-[β-D-glucopyranosyl-(1→6)-β-D-glucopyranoside]	194
Curculigenin	155
Isocurculigenin	131
Cycloartane glycoside (5)	30

**Table 2:** The molecule-target protein pairs for molecular docking.

Molecule	Target gene name
4', 5, 7-trihydroxyflavone	FOS
4', 5, 7-trihydroxyflavone	SERPINE1
Ethyl protocatechuate	SERPINE1
3, 5-dihydroxy-benzoic acid	SERPINE1
4', 5, 7-trihydroxyflavone	AKR1C3
Curculigosaponin A	AKR1C3
Curculigine G	AKR1C3
Curculigosaponin A	FGF2
Cycloartane glycoside (5)	FGF2

## Abbreviations

*D. indica*: *Duchesnea indica*
HCC: hepatocellular carcinoma
DEG: differentially expressed genes
TCM: traditional Chinese medicine
MEM: minimal essential medium
FBS: fetal bovine serum
MS: mass spectrometry
GSEA: Gene Set Enrichment Analysis
PPI: protein-protein interaction
HMDB: Human Metabolome Database
TCMSP: Traditional Chinese Medicine Systems Pharmacology Database and Analysis Platform
DC: degree centrality
BP: biological process
FDR: false discovery rate
FC: fold change
