# Supplementary material for: Network Pharmacology Combined with Experimental Validation Reveals the Anti-tumor Effect of Duchesnea indica against Hepatocellular Carcinoma
Source: J Cancer. 2023 Feb 13;14(4):505–18. doi: 10.7150/jca.76591 (PMC10088531; doi:10.7150/jca.76591)
Supplement: Supplementary file 1 — Supplementary figures and table. [file jcav14p0505s1.pdf]

Supplementary materials

Supplementary Figures

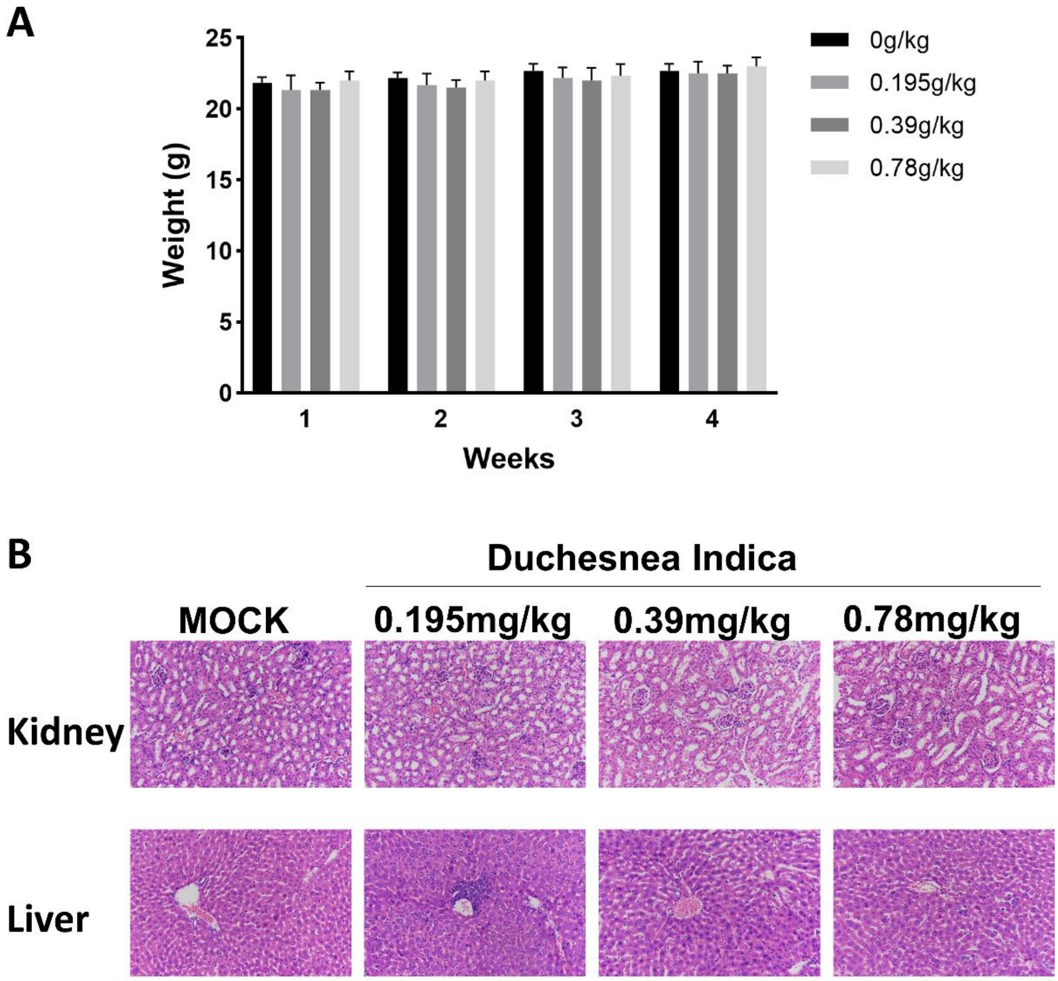

Figure S1. Evaluation of side effects of *D. indica* on HCC mice. (A) *D. indica* has no effect on the weight of mice. (B) *D. indica* has no effect on kidney and liver morphology.

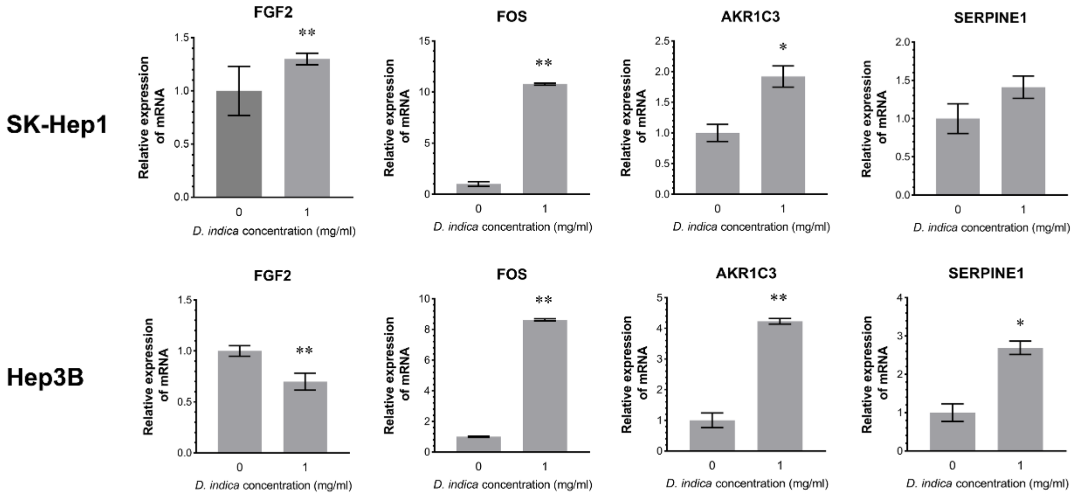

Figure S2. The expression levels of FOS, SERPINE1, AKR1C3, and FGF2 in SK-Hep1 and Hep3B cells with and without D. indica administration. *, $p < 0.05$; **, $p < 0.01$.

Supplementary Table

Table S1 The primer sequences.

Gene symbol	Forward primer	Reversed primer	length
**FGF2**	AGTGTGTGCTAACC GTTACCT	ACTGCCCAGTTCG TTTCAGTG	170
**Fos**	CACTCCAAGCGGAG ACAGAC	AGGTCATCAGGGA TCTTGCAG	139
**AKR1C3**	TCTGGGATCTCAAC GAGACAA	TGGAACTCAAAAA CCTGCACG	207
**SERPINE1**	AGTGGACTTTTCAG AGGTGGA	GCCGTTGAAGTAG AGGGCATT	151
**ACTB**	AGAGCCTCGCCTTT G	CATAGGAATCCTT CTGACC	
